# The Association Between Age and Prognosis in Patients Under 45 Years of Age With Anti-NMDA Receptor Encephalitis

**DOI:** 10.3389/fneur.2020.612632

**Published:** 2020-12-29

**Authors:** Yueqian Sun, Guoping Ren, Jiechuan Ren, Wei Shan, Xiong Han, Yajun Lian, Tiancheng Wang, Qun Wang

**Affiliations:** ^1^Department of Neurology, Beijing Tiantan Hospital, Capital Medical University, Beijing, China; ^2^China National Clinical Research Center for Neurological Diseases, Beijing, China; ^3^Beijing Institute for Brain Disorders, Beijing, China; ^4^Department of Neurology, Henan Provincial People's Hospital, Zhengzhou, China; ^5^Department of Neurology, The First Affiliated Hospital of Zhengzhou University, Zhengzhou, China; ^6^Department of Neurology, Lanzhou University Second Hospital, Lanzhou, China

**Keywords:** anti-NMDA receptor encephalitis, prognosis, age, gender, modified Rankin Scale

## Abstract

This study aims to evaluate the association between age and prognosis in patients with anti-N-methyl-D-aspartate receptor encephalitis (anti-NMDARE) under the age of 45 years. A retrospective study was conducted in patients younger than 45 years diagnosed as anti-NMDARE in four hospitals in China. Age at admission was divided into four categories: <15, 15–24, 25–34, 35–45 years. Neurological prognosis was evaluated using modified Rankin Scale. Adjusted multivariable logistic regression was used to analyze the association. The multivariable-adjusted odds ratios (95% confidence interval) of prognosis in anti-NMDARE across the categories of age were as follows: in males, 1.00 (reference), 4.76 (0.39–58.76), 13.50 (0.79–230.40), and 8.81 (0.36–218.39) (*P* for trend = 0.171); in females, 1.00 (reference), 7.27 (0.36–146.19), 20.08 (1.09–370.39), and 54.41 (1.60–1,849.10) (*P* for trend = 0.01). We concluded that the increasing age was associated with a poorer prognosis of anti-NMDARE in females but not males.

## Introduction

Anti-N-methyl-D-aspartate receptor encephalitis (anti-NMDARE) is an autoimmune disease associated with a broad range of complex neuropsychiatric symptoms and the presence of antibodies in cerebrospinal fluid (CSF) bind to the GluN1 subunit of the NMDAR (1). It is the most frequently recognized neuronal-antibody-mediated encephalitis (2, 3). Anti-NMDARE is more likely to occur on those who are of younger ages (median age at disease onset is 21 years, range: <1–85 years) and a multicenter observational study showed 95% of 577 patients were younger than 45 years (4). Anti-NMDAR antibodies may be detectable in up to 1% of young patients with encephalitis of unknown etiology admitted to the intensive care unit (ICU) (2) and about 70% of anti-NMDARE patients are admitted to ICU (4, 5). Various curative interventions have been proposed and advocated, and first line treatment includes methylprednisolone pulse therapy, intravenous immunoglobulin (IVIG) and plasmapheresis (PLEX). And if an identified malignancy such as a teratoma is found, surgical removal should be performed. Although many patients respond well to immunotherapy and (when needed) tumor removal, 20% do not achieve remission upon therapy (4). A better understanding of the factors affecting prognosis may inform patients and their families about likely outcomes and guide clinicians' treatment decisions. Data concerning the correlation between age and prognosis remain scarce and inconsistent. Several studies (6, 7) have documented that the outcome is poorer in older patients, while other studies (8, 9) showed no a clear association between age and prognosis. In light of this, we undertook a retrospective study to provide a more comprehensive conclusion of the relationship between age and prognosis.

## Materials and Methods

### Study Design and Participants

This retrospective study was reviewed, approved by The Research Ethics committee of Beijing Tiantan Hospital, Henan Provincial People's Hospital, the First Affiliated Hospital of Zhengzhou University, and Second Hospital of Lanzhou University between January 2015 and June 2019. The study used data from the aforementioned ethics committee.

### Data Collection

Patients aged under 45 years who met criteria for definite anti-NMDARE (10) were included, and those suspecting of other autoimmune disorder or with missing key clinical data or mRS > 0 before the onset of symptoms were excluded.

The following factors were collected: (1) demographics (sex and age); (2) clinical features [viral prodrome (headache, fever, nausea and vomiting), disturbance of consciousness, abnormal behavior, memory impairment, speech disorders, insomnia, seizures, autonomic dysfunction and movement disorders]; (3) laboratory and radiographic findings [CSF protein and white blood cell (WBC) count and brain magnetic resonance imaging (MRI)]; (4) other clinical features [diagnosis of tumor, immunotherapy latency (the time interval from onset to the initiation of immunotherapy)]; (5) modified Rankin Scale (mRS). MRI scans were classified as abnormal based on T2-weighted or fluid-attenuated inversion recovery (FLAIR) hyperintensity in one or both medial temporal lobes, multiple inflammation or demyelination involving gray and white matter (10). Neurological outcomes were evaluated using mRS (11). And mRS ≤ 2 was defined as “good prognosis,” whereas > 2 as “poor prognosis.” We divided subjects into four different age groups (<15, 15–24, 25–34, 35–45 years) to assess outcome variation by age.

### Statistical Analysis

Continuous variables were tested for normal distribution using the Shapiro-Wilk (*n* ≤ 2,000) tests. The variables displayed normal distributions and were presented as the mean ± standard deviation (SD), while those for categorical variables were expressed as number and percentage. To compare variables between subgroups of patients, we used Mann-Whitney U tests for continuous variables and Chi-square tests for categorical variables. Variables with a *p*-value below 0.20 on univariate analysis were included in multivariate logistic regression, which was used to describe the odds ratio (OR) between age and prognosis, after adjustment for potential confounding factors. The differences were considered to be statistically significant at *p* < 0.05. SPSS 23.0 software was used for statistical analyses. Data were all expressed as OR with 95% confidence interval (CI).

## Results

A total of 108 participants were enrolled in this study, half of whom were males, with mean (SD) age of 23.21 (9.50) years and half of whom were females, with mean (SD) age of 27.74 (8.95) years. The status of age number in categories (<15, 15–24, 25–34, 35–45 years) were, respectively, 18.5, 35.2, 34.3, and 12.0%. The poor prognosis of anti-NMDARE patients under 45 years of age was 31.5% (34 of 108) (29.6 and 33.3% for males and females, respectively).

Consciousness disorder was more commonly seen in the poor prognosis group than that in the good prognosis group (*p* = 0.003). And the difference of movement abnormalities was also statistically significant between the two groups (*p* = 0.016). No other differences were statistically significant (Table 1).

**Table 1:** Baseline characteristics of 108 patients of anti-NMDARE enrolled in the study.

**Factor**	**mRS ≤ 2**	**mRS > 2**	***P*-value**
Number of cases	74	34	
Gender			0.679
1	38 (51%)	16 (47%)	
2	36 (49%)	18 (53%)	
Age			0.127
<15	17 (23%)	3 (9%)	
15–24	28 (38%)	10 (29%)	
25–34	22 (30%)	15 (44%)	
35–45	7 (9%)	6 (18%)	
Memory dysfunction			0.241
0	52 (70%)	20 (59%)	
1	22 (30%)	14 (41%)	
Speech disorders			0.24
0	54 (73%)	21 (62%)	
1	20 (27%)	13 (38%)	
Behavioral changes			0.123
0	31 (42%)	9 (26%)	
1	43 (58%)	25 (74%)	
Seizures			0.458
0	25 (34%)	14 (41%)	
1	49 (66%)	20 (59%)	
Consciousness disorder			0.003
0	63 (85%)	20 (59%)	
1	11 (15%)	14 (41%)	
Sleep dysfunction			0.579
0	62 (84%)	27 (79%)	
1	12 (16%)	7 (21%)	
Movement disorder			0.016
0	58 (78%)	19 (56%)	
1	16 (22%)	15 (44%)	
Autonomic dysfunction			0.131
0	71 (96%)	30 (88%)	
1	3 (4%)	4 (12%)	
Viral prodrome			0.145
0	35 (47%)	11 (32%)	
1	39 (53%)	23 (68%)	
CSF WBC count (>20 cell/μL)			0.105
0	45 (61%)	15 (44%)	
1	29 (39%)	19 (56%)	
CSF protein (>30 mg/dL)			0.914
0	34 (46%)	16 (47%)	
1	40 (54%)	18 (53%)	
Abnormal MRI			0.761
0	28 (38%)	12 (35%)	
1	45 (62%)	22 (65%)	
Tumor			0.571
0	70 (95%)	33 (97%)	
1	4 (5%)	1 (3%)	
Immunotherapy latency (>4 wk)			0.077
0	44 (59%)	14 (41%)	
1	30 (41%)	20 (59%)	

*Data presented as n (%)*.

*mRS, modified Rankin Scale; anti-NMDARE, anti-N-methyl-D-aspartate receptor encephalitis; CSF, cerebrospinal fluid; WBC, white blood cell; MRI, magnetic resonance imaging*.

*Univariate binary logistic regression analysis was conducted using the Fisher exact test; the P-value was derived from bivariate association analyses between each study variables and the mRS score*.

Multiple logistic regression models examined factors associated with poor prognosis (Table 2). In the multivariate analysis, all of the factors with a *P*-value < 0.20 in Table 1 were added into the final model. It is evident that increasing age was associated with inferior outcome. Age (*p* = 0.002) was shown to be independent prognostic factors for anti-NMDARE. It also showed that behavioral disorders (OR = 4.27; 95% CI, 1.25–14.57), disturbances in consciousness (OR = 7.73; 95% CI, 2.29–26.12), movement abnormalities (OR = 7.13; 95% CI, 2.14–23.76), autonomic dysfunction (OR = 0.82; 95% CI, 0.12–5.44), viral prodrome (OR = 2.30; 95% CI, 0.74–7.13), abnormal WBC count (OR = 3.74; 95% CI, 1.16–12.04), and immunotherapy latency (OR = 5.17; 95% CI, 1.44–18.53) were independent risk factors associated to the prognosis of participants with anti-NMDARE under 45 years of age (Table 2).

**Table 2:** Multivariate analysis of factors associated with poor prognosis in patients with anti-NMDARE.

**Variable and Intercept**	**β Coefficient**	**OR (95%CI)**	***P*-value**
Age	0.096	1.10 (1.04–1.17)	0.002
Behavioral changes	1.451	4.27 (1.25–14.57)	0.021
Consciousness disorder	2.046	7.73 (2.29–26.12)	0.001
Movement disorder	1.964	7.13 (2.14–23.76)	0.001
Autonomic dysfunction	−0.196	0.82 (0.12–5.44)	0.839
Viral prodrome	0.831	2.30 (0.74–7.13)	0.151
CSF WBC count (>20 cell/μL)	1.319	3.74 (1.16–12.04)	0.027
Immunotherapy latency (>4 wk)	1.642	5.17 (1.44–18.53)	0.012
Intercept	−7.359	NA	NA

*NA, not applicable; anti-NMDARE, anti-N-methyl-D-aspartate receptor encephalitis; CSF, cerebrospinal fluid; WBC, white blood cell; mRS, modified Rankin Scale*.

Table 3 shows crude and adjusted association between the category of age and poor prognosis. We found a statistically significant age-gender interactions (*p* = 0.017). The multivariable-adjusted odds ratios (95% CI) in anti-NMDARE patients below the age of 45 of poor prognosis throughout the age categories were as follows: in males, 1.00 (reference), 4.76 (0.39–58.76), 13.50 (0.79–230.40), and 8.81 (0.36–218.39) (*P* for trend = 0.171); in females, 1.00 (reference), 7.27 (0.36–146.19), 20.08 (1.09–370.39), and 54.41 (1.60–1,849.10) (*P* for trend = 0.01) (Table 3). No other significant differences were found among the four age categories.

**Table 3:** Adjusted odds ratios (with 95% confidence intervals) from logistic regression models for associations of age with poor prognosis.

	**Age**				***P* for trend**
	**<15**	**15–24**	**25–34**	**35–45**	
**MALE**					
No. of subjects	10	19	18	7	–
Poor prognosis	2	6	6	2	–
Model 1	1.00 (reference)	3.68 (0.39, 34.47)	7.07 (0.62, 80.54)	4.44 (0.29, 68.20)	0.271
Model 2	1.00 (reference)	4.76 (0.39, 58.76)	13.50 (0.79, 230.40)	8.81 (0.36, 218.39)	0.171
**FEMALE**					
No. of subjects	10	19	19	6	–
Poor prognosis	1	4	9	4	–
Model 1	1.00 (reference)	4.64 (0.33, 65.01)	13.14 (0.98, 177.06)	46.82 (1.99, 1100.83)	0.006
Model 2	1.00 (reference)	7.27 (0.36, 146.19)	20.08 (1.09, 370.39)	54.41 (1.60, 1849.10)	0.01

*Model 1, Adjusted for disturbances in consciousness and movement abnormalities; Model 2, Adjusted for disturbances in consciousness, movement abnormalities, abnormal WBC count and immunotherapy latency*.

## Discussion

**Figure 1:**
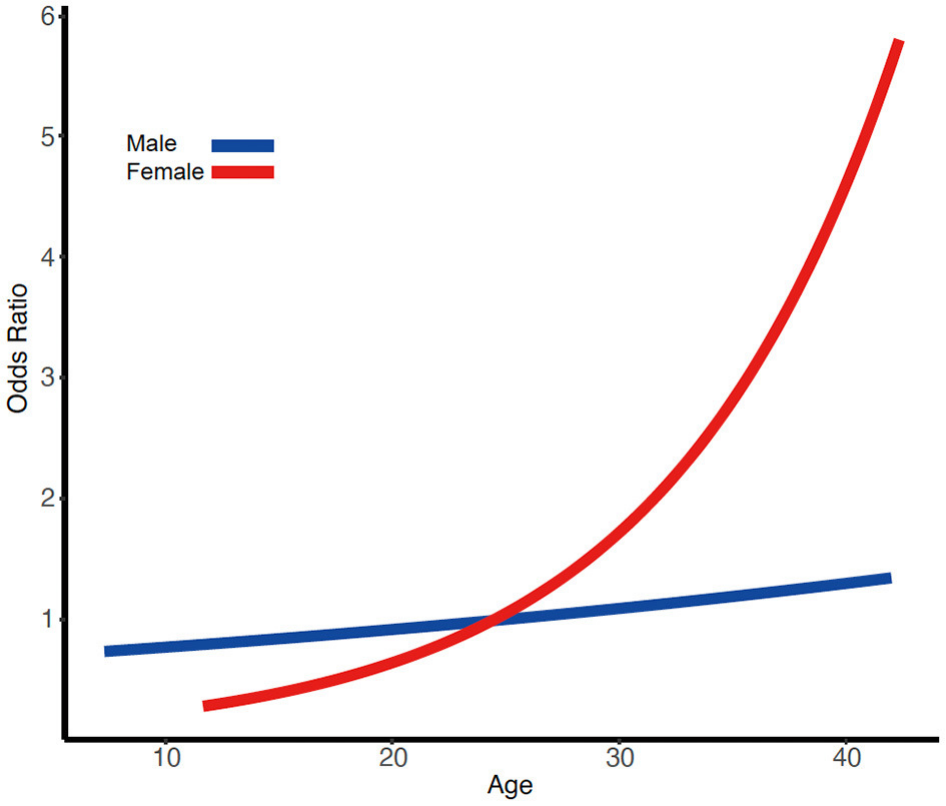
Illustration of the pattern of results from the multiple linear regression analysis of poor prognosis. Risk of poor prognosis increases more with age for women than for men.

Our findings showed that poor prognosis tended to occur at females with older age than younger demographic in anti-NMDARE patients below the age of 45, after adjustment for confounding factors (Table 3 and Figure 1). In contrast, there was no association between age and prognosis in males. As far as we are aware, this is the first study to directly address the association between age and prognosis among patients with anti-NMDARE aged <45 years.

Previous studies (12, 13) have showed that prognosis was related to consciousness disorder, we therefore did adjust for this variable at first. The association between age and prognosis was significantly affected by this adjustment, which indicated that consciousness disorder could be considered as a major confounding factor. This may be at least partially due to increased risk of severe pneumonia, multiple organ failure or other life-threatening complications. And also, it might contribute to the delayed diagnosis and treatment and in turn have an impact on prognosis. We have subsequently adjusted for factors associated with prognosis including movement abnormalities, abnormal WBC count and immunotherapy latency, which have already been reported to be associated with poor prognosis (9). Surprisingly, a more pronounced association was found in females after these adjustments.

Consistent with previous studies (7, 12), our results revealed that the older the patients, the worse the prognosis. Nevertheless, using age as a categorical variable as opposed to a continuous measure, earlier studies failed to fully describe the association between age and prognosis. Hence, we decided to analyze age as a continuous variable, which allowed us to avoid any kind of classification bias and to identify age as an independent risk factor. A negative linear correlation between age and prognosis was found. This finding will cast light on the impact of age on prognosis in anti-NMDARE aged younger than 45 years and ultimately offer the individualized risk assessment of therapy among the diverse age groups. Additionally, our assessment of confounding factors—such as disturbance of consciousness—may enlarge our knowledge and understanding of the actual correlations between the age and prognosis in anti-NMDARE below the age of 45.

There could be multiple reasons for present results. First, it is widely accepted that individuals with older age are prone to organ dysfunction, decreased immunity and other systemic diseases. And also, delays in diagnosis and treatment are more frequent among those patients (6). Put another way, due to multiple comorbidities, clinical preconceptions and variety of clinical presentations, an accurate medical care for older patients is often delayed (14). The time since onset to immunotherapy is a known predictor in anti-NMDARE, which signifies that the earlier diagnosed and treated, the better the outcome. This may be a cause of poor prognosis in elderly patients. In addition, the older population is at a greater risk of carcinoma (6, 15), which may be one of the contributors to the poor prognosis. Besides, the ultimate goal of immunotherapy is to stimulate immune responses, and poor immune responsiveness among the elderly results in suboptimal responses to immunization.

Several explanations for the gender-specific association can be considered. As we all know, anti-NMDARE has been identified to have a significant correlation with ovarian teratomas. Teratoma affects up to 60% of adult women, but rarely occurs in males (16). It has been classified that tumor removal benefited neurologic prognosis in tumor related anti-NMDARE (15).

## Limitations

Several limitations qualify our findings. First, we included the cases registered with positive antibody, not patients eventually diagnosed with probable anti-NMDARE not meeting criteria for a definite anti-NMDARE category. Second, generalizability of findings is constrained. We looked only at patients who were under 45 years old, thus, the present results are only applicable to similar populations and cannot be extrapolated to different populations. Third, despite adjusting for a comprehensive set of potential confounders, the results could be potentially biased by residual confounding we cannot account for. In addition, there was no long-term functional status data after discharge in our analysis, which does not allow for conclusions on long-term prognosis. Further prospective studies with larger sample size are needed.

## Conclusion

Our findings on patients under 45 years of age with anti-NMDARE revealed that increasing age as a continuous variable has a negative impact on the mRS score at discharge in females, but not in males. We further explored the possible underlying mechanisms. The present study aid with affording medical evidence of therapies at an early stage for patients with anti-NMDARE under 45 years of age. Further studies with larger sample sizes are needed to further verify these associations.

## Data Availability Statement

The original contributions presented in the study are included in the article/supplementary material, further inquiries can be directed to the corresponding author/s.

## Author Contributions

QW concepted, designed, and supervised the study. YS, XH, YL, and TW acquired the data. YS analyzed and interpreted the data, provided statistical analysis, had full access to all of the data in the study, and are responsible for the integrity of the data and the accuracy of the data analysis and drafted the manuscript. GR, JR, and QW critically revised the manuscript for important intellectual content. All authors contributed to the article and approved the submitted version.

## Conflict of Interest

The authors declare that the research was conducted in the absence of any commercial or financial relationships that could be construed as a potential conflict of interest.
